# A Case of Acalvaria in a Full Term, Live Born Male Infant

**DOI:** 10.7759/cureus.22430

**Published:** 2022-02-21

**Authors:** Felistia N Crowder, Seth J Deskins, Morgan Decker, Kylie Parrish

**Affiliations:** 1 Pediatrics, West Virginia University Health System, Morgantown, USA; 2 Internal Medicine-Pediatrics, West Virginia University Health System, Morgantown, USA

**Keywords:** exencephaly, anencephaly, acrania, acalvaria, cranial vault defect

## Abstract

Acalvaria is a rare cranial vault defect characterized by the presence of the cerebellum and cerebral cortex with the absence of the calvarium above the orbits, intact facial structures, and the presence of dura mater. Unfortunately, this diagnosis comes with a dim prognosis that is not compatible with life long-term. First-trimester diagnosis with ultrasonography can establish the diagnosis. If imaging is equivocal, advanced imaging with fetal MRI has a role to aid in distinguishing between similar cranial vault defects that fall on the spectrum including anencephaly, exencephaly, and acrania. We present the case of a term male infant with known acalvaria diagnosed incidentally on prenatal ultrasound that was delivered by cesarean section to a G3P3 mother at the time of delivery with two prior uncomplicated pregnancies. Maternal history was rather insignificant except for gestational diabetes and gestational hypertension well-controlled without medication. After initiation of comfort measures only, the infant expired on day of life two.

## Introduction

Cranial vault defects comprise a spectrum of rare conditions with generally poor prognoses that are typically diagnosed on prenatal ultrasound in the first trimester. These conditions include exencephaly, anencephaly, acrania, and acalvaria. As described in the following case, acalvaria is defined as deformed brain tissue with the absence of the calvarium above the orbits but intact facial structure and skull base as well as dura mater. We present a case of acalvaria discovered early in the first trimester where the fetus was carried to term and expired on day of life two.

## Case presentation

A male infant was born at 37 weeks and 1 day to a 28-year-old G3P3 female via scheduled cesarean section for a known congenital cranial vault defect discovered incidentally on prenatal ultrasound. The neonatal intensive care unit (NICU) team was in attendance for the delivery where no resuscitation was required. APGARs were eight and eight at one and five minutes of life. The infant was noted to have spontaneous respirations, weak cry, and exposed brain tissue with minimal bleeding noted from the posterior brain. Comfort measures only were initiated after delivery. The family periodically attempted feeds for comfort. The patient received a few doses of morphine for suspected pain with head dressing changes and had seizure-like activity consisting of eye-rolling and tremors. The infant expired on day of life two. The family requested an autopsy and cytogenetics to be performed. The autopsy revealed marked malformations on gross examination that were deemed secondary to “disruptive influences of neural development in the face of acrania” while neural tube closure seemed to be present. The report also noted that the “presence of amniotic membrane in the apparent dura mater samples raises the possibility of acrania due to an amniotic band.” Other findings on autopsy included cryptorchidism, patent foramen ovale, and patent ductus arteriosus. Cytogenetic testing was normal.

**Figure 1:**
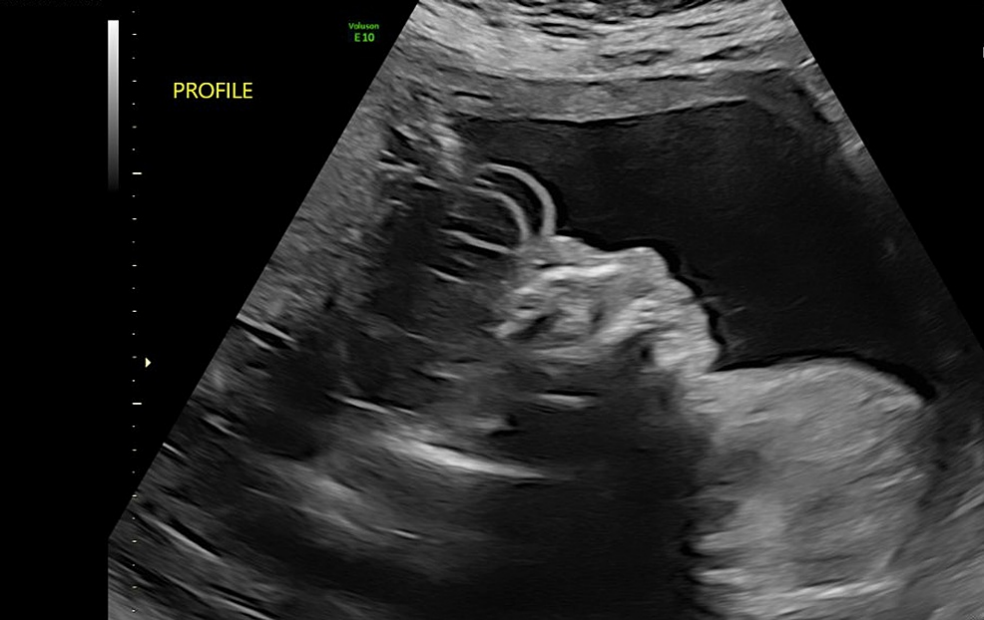
Ultrasound at 23 weeks noting: “acalvaria with an absence of the parietal, temporal, and occipital bones but with skull base and facial bones intact; disorganized brain tissue is seen floating in the amniotic fluid.”

**Figure 2:**
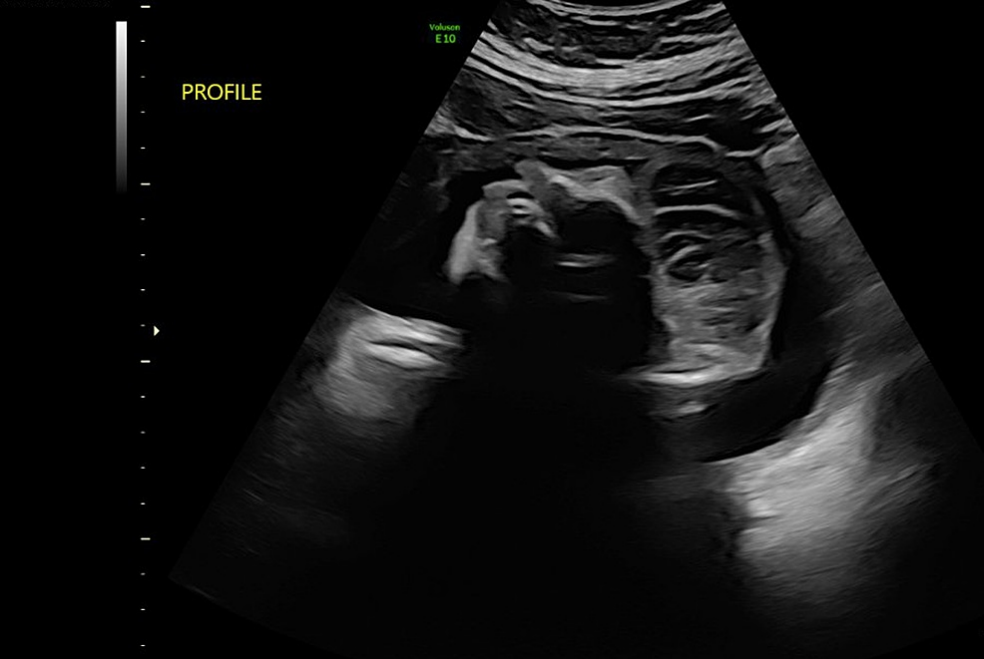
Ultrasound at 30 weeks noting: “acalvaria again noted – the skull bones are absent from the level of the orbit up, there is disorganized brain tissue floating in the amniotic fluid.”

Regarding maternal history, the mother was referred to our facility by an outside provider after a prenatal ultrasound was concerning a major fetal brain/skull defect suspicious for acrania. An ultrasound at 19 weeks at our facility confirmed the presence of a defect and was read as “brain tissue visualized but does not appear to be organized into recognizable components.” The mother declined amniocentesis and termination of the pregnancy, as she desired to carry the infant to term. Repeat ultrasound at 23 weeks gestation (Figure 1) revealed “acalvaria with an absence of the parietal, temporal, and occipital bones but with skull base and facial bones intact; disorganized brain tissue is seen floating in the amniotic fluid.” Acalvaria was again noted in the ultrasound at 30 weeks gestation (Figure 2).

Cell-free DNA was significant for a low-risk XY fetus, and the carrier screen was negative. Prenatal lab screening was significant for equivocal rubella status along with negative hepatitis B, hepatitis C, human immunodeficiency virus (HIV), syphilis via venereal disease research laboratory test (VDRL), gonorrhea/chlamydia, and group B streptococcus (GBS). There was no known history of SARS-CoV-2 infection during pregnancy. Maternal blood type was O negative. Other maternal history was significant for obesity and gestational diabetes and gestational hypertension that were both well-controlled without medical intervention. The mother experienced frequent urinary tract infections during the pregnancy requiring two courses of antibiotics, one of them being nitrofurantoin. Other medications during pregnancy included a prenatal vitamin, iron supplement, and pantoprazole for acid reflux. The mother was a former smoker with a quit date during the course of the pregnancy. She reported no drug use, and confirmation urine drug screen was negative. There was no family history of genetic conditions or bleeding or clotting disorders. Prior pregnancies were uncomplicated and resulted in two full-term healthy births.

## Discussion

Congenital cranial vault defects refer to a group of conditions including exencephaly, anencephaly, acrania, and acalvaria all of which are associated with a dismal prognosis [1,2]. The technical differences with the terminology are rather confusing, which can create difficulty in making a definitive diagnosis prenatally. Exencephaly is an incredibly rare malformation with a large amount of protruding disorganized brain tissue covered only by a vascular layer of the epithelium [1,3]. It is defined by a normal skull base with intact facial structure and absence of the calvarium and scalp above the orbits [1]. Exencephaly is thought to be the embryological precursor to anencephaly [3]. Anencephaly is distinct from exencephaly in the fact that the brain and calvarium are both absent above the orbits [4]. Anencephaly typically is associated with deformed facial structures as well [4]. Acrania is defined by the complete absence of the neurocranium with abnormally developed brain present, which is covered by a membrane [1]. Finally, acalvaria, as described in our case, is defined by the absence of the calvarial bones but with preserved skull base, facial bones, and present but deformed cerebral hemispheres that are covered with dura and skin [1,4]. While the ultrasound reads and autopsy results used differing terminology in our case, the final diagnosis was acalvaria given the presence of dura mater noted at autopsy, absence of calvarial bones, and preserved skull base and facial bones. This further emphasizes how challenging the final diagnosis can be due to the nuances of different cranial vault defects.

Diagnosis of any of the cranial vault defects can be established by ultrasound in the first trimester of pregnancy [5]. Fetal ultrasound can be equivocal in the diagnosis especially earlier in the course, and fetal MRI has become an increasingly utilized tool for confirmatory diagnosis when ultrasound results are indeterminate [1]. The importance of accurately defining the condition and determining the correct nomenclature to use while the fetus is in utero is critical for appropriate parental counseling, which often will include high-risk counseling with a maternal-fetal medicine specialist [4,5]. It is important to note that no chromosomal abnormalities have been linked to acalvaria, with most cases likely being caused by sporadic events in utero [4]. The prognosis of cranial vault defects, regardless of the exact malformation, remains poor with a large majority of pregnancies resulting in stillborn births [1,3]. Early recognition is critical in helping parents make an informed decision regarding the continuation of pregnancy.

## Conclusions

We presented the rare case of an infant born with acalvaria diagnosed incidentally on prenatal ultrasound who expired on day of life two. This case demonstrates the difficulties in making an exact diagnosis prenatally due to the nuances of various cranial default defects and the rarity of cases. No known genetic or chromosomal abnormalities have been linked to acalvaria. Due to the poor prognosis associated with cranial vault defects, it is especially important to identify these abnormalities prenatally in order to provide appropriate counseling to parents.
